# A pan-European dataset revealing variability in lithic technology, toolkits, and artefact shapes ~15-11 kya

**DOI:** 10.1038/s41597-023-02500-9

**Published:** 2023-09-07

**Authors:** Shumon T. Hussain, Felix Riede, David N. Matzig, Miguel Biard, Philippe Crombé, Javier Fernández-Lopéz de Pablo, Federica Fontana, Daniel Groß, Thomas Hess, Mathieu Langlais, Ludovic Mevel, William Mills, Martin Moník, Nicolas Naudinot, Caroline Posch, Tomas Rimkus, Damian Stefański, Hans Vandendriessche

**Affiliations:** 1 Department of Archaeology and Heritage Studies, Aarhus University, Moesgård Allé 20, 8270 Højbjerg, Denmark; 2 INRAP, Centre Île-de-France, Institut National de Recherches Archéologiques Préventives, 18 rue Chapelle, 89510 PASSY/UMR 8068 TEMPS, Technologie et Ethnologie des Mondes Préhistoriques, Paris, Nanterre France; 3 Department of Archaeology, Ghent University, Sint-Pietersnieuwstraat 35, 9000 Ghent, Belgium; 4 I.U. de Investigación en Arqueología y Patrimonio Histórico, University of Alicante, Alicante, Spain; 5 Dipartimento di Studi Umanistici – Sezione di Scienze Preistoriche e Antropologiche, University of Ferrara, Ferrara, Italy; 6 Museum Lolland-Falster, Frisegade 40, 4800 Nykøbing F, Denmark; 7 CNRS UMR 5199 PACEA, University of Bordeaux, Bordeaux, France; 8 CNRS UMR UMR 8068 TEMPS, Paris, Nanterre France; 9 ZSBA, Schloß Gottdorf, Schloßinsel 1, 24837 Schleswig, Germany; 10 Department of Geology, Faculty of Science, Palacky University Olomouc, Olomouc, Czech Republic; 11 Université Côte d’Azur, UMR 7264 CEPAM, CNRS, Nice, France; 12 Natural History Museum Vienna, Burgring 7, 1010 Vienna, Austria; 13 Institute of Baltic Region History and Archaeology, Klaipėda University, Klaipėda, Lithuania; 14 Archaeological Museum in Kraków, Kraków, Poland

**Keywords:** Archaeology, Databases

## Abstract

Comparative macro-archaeological investigations of the human deep past rely on the availability of unified, quality-checked datasets integrating different layers of observation. Information on the durable and ubiquitous record of Paleolithic stone artefacts and technological choices are especially pertinent to this endeavour. We here present a large expert-sourced collaborative dataset for the study of stone tool technology and artefact shape evolution across Europe between ~15.000 and 11.000 years before present. The dataset contains a compendium of key sites from the study period, and data on lithic technology and toolkit composition at the level of the cultural taxa represented by those sites. The dataset further encompasses 2D shapes of selected lithic artefact groups (armatures, endscrapers, and borers/perforators) shared between cultural taxa. These data offer novel possibilities to explore between-regional patterns of material culture change to reveal scale-dependent processes of long-term technological evolution in mobile hunter-gatherer societies at the end of the Pleistocene. Our dataset facilitates state-of-the-art quantitative analyses and showcases the benefits of collaborative data collation and synthesis.

## Background & summary

The Late Glacial (c. 14.7-11.6 ka BP) is a pivotal period in European prehistory. It links the later part of the Weichselian Pleniglacial (c. 20-14.7 ka BP) – the supposed apogee of Upper Paleolithic techno-cultural efflorescence – with the subsequent Holocene (c. 11.8 ka BP-today) and its diverse woodland forager, pastoral and early agricultural societies. Situated at the Pleistocene-Holocene juncture and characterized by a series of volatile but high resolution environmental and climatic upheavals, the Late Glacial has been a central arena of scholarly debate on the impact of climate change on human societies^1–5^, on palaeodemography^6–10^, the evolution of so-called ‘complex’ or ‘transegalitarian’ hunter-gatherers^11,12^, and the diversification of human lifeways and adaptations that preceded post-Pleistocene trajectories of cultural evolution^13,14^. By the same token, this period has also been stereotyped as documenting cultural loss and technological simplification^15^. Increasingly, ancient genomic data is similarly informing interpretations of movement and contact in this period^9,16^, yet many of these interpretations remain difficult to substantiate given the lack of relevant large-scale comparative archaeological data which can be readily mobilized to test competing hypotheses or to explore scale-dependent spatiotemporal dynamics of within-period change above the level of sites or regions. Yet, due to its comparatively well-resolved human and environmental archives and limited recovery bias compared to older periods, European Late Glacial archaeology harbors the potential to benchmark hunter-gatherer research in other periods of the human deep past. To do so, information on stone artefact variability, production technologies and design choices (henceforth: lithic data) is vital for our understanding of early human evolution as stone artefacts are abundant, durable and yield rich insights into fundamental dimensions of human behavior^17,18^.

We here describe an open-access dataset^19^ compiled by a concerted effort of lithic experts across Europe to help overcoming this problem and to begin asking pan-European questions on synchronic and diachronic techno-cultural variability, and to facilitate critical interrogations of the scope and quality of current knowledge and the significance of higher-level taxonomic denominations to organize the record (e.g. Final Magdalenian, Hamburgian, Azilian, Federmesser, Ahrensburgian, Swiderian, Belloisian, etc.)^20,21^. The dataset^19^ was constructed with the aim of 1) rendering long-standing hypotheses and narrative framings of the Late Glacial amendable to systematic empirical scrutiny, and 2) breaking away from terminological and semantic discussions and instead to open new possibilities for data-driven investigations of millennial-scale patterns and trends in human technological behavior beyond labels and categorizations largely contingent on research history. Two complementary but interrelated concerns motivate our effort: a) the growing recognition of scale in archaeology and cultural evolutionary studies with the resulting need to take serious hitherto largely neglected macro-archaeological processes and phenomena^22,23^, and b) the impeding effects of regional research traditions and culture-historical frames of reference for regional and continental-scale comparison^20,24^, to address both cross-sectional and longitudinal questions in Late Glacial archaeology and beyond. Our efforts resonate with calls for synthesis and higher-level archaeological analysis^21,25^ using ‘big data’ or at least unprecedentedly large datasets, while also duly accounting for the fragmentary and complex nature of the available material registers^26–28^. The latter draws attention to a nascent transformation in knowledge ecologies based on an ethos of sharing and collaborative inquiry^25,29–31^ necessary to develop data infrastructures promoting broad-scale comparative investigations at cross-regional scales. The here-presented dataset^19^ is a first attempt to work towards such a shared data infrastructure for Late Glacial Europe, to build a scalable higher-order data framework for lithic analysts and to probe into the untested utility and potential of such integrated collaborative work. In contrast to previous database projects in Pleistocene archaeology^32,33^, our point of departure is not individual sites and the lithic assemblages they host, but amalgamated higher-order units which reflect the current state of research in different European regions, allowing us to transparently assemble data on different lithic domains operating on varying scales of observation, in turn offering unique possibilities of archaeological analysis and comparison.

## Methods

### Taxonomic units, macro-regions and time slices

**Fig. 1:**
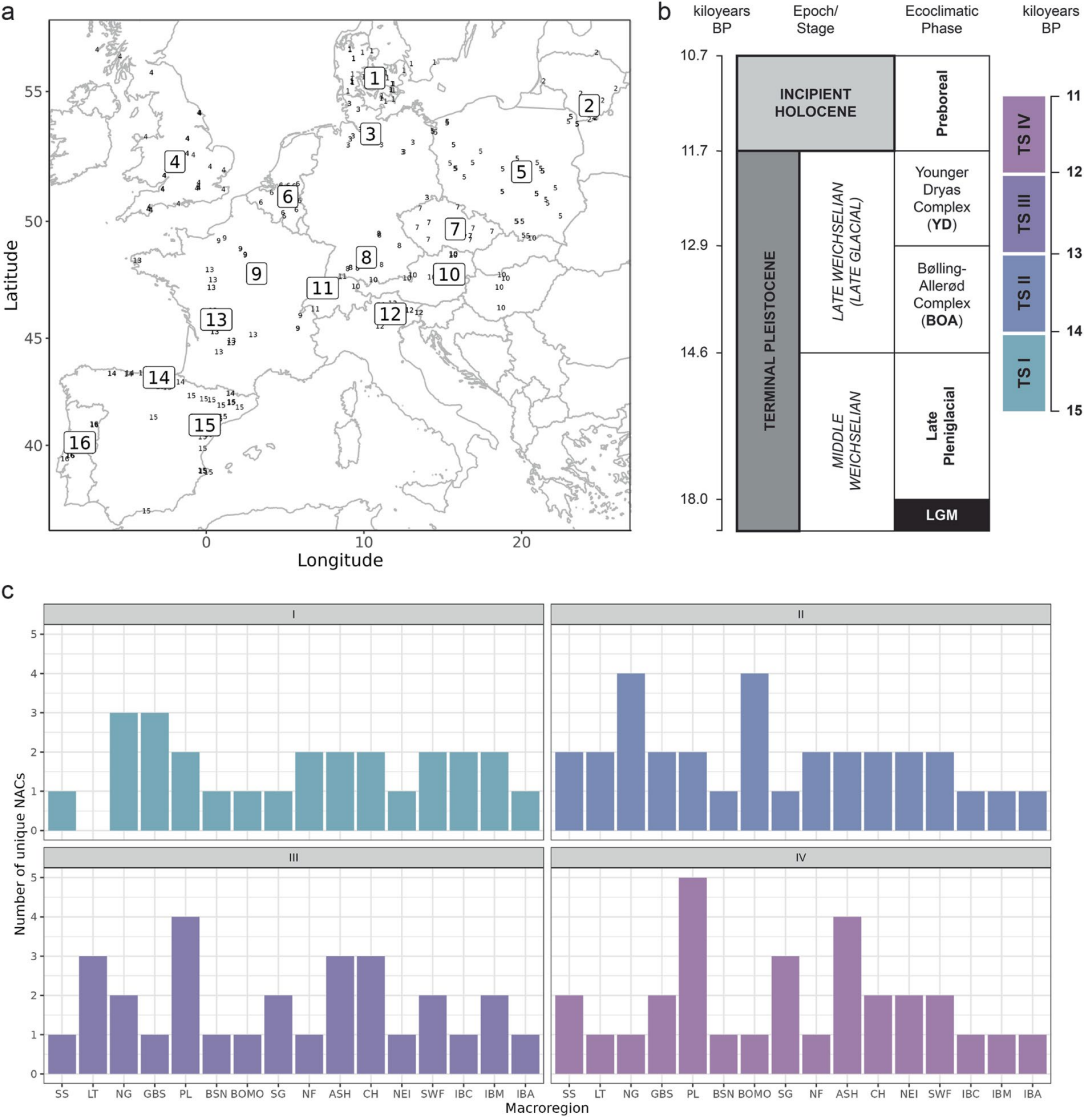
Spatiotemporal characteristics of the dataset^19^. (**a**) Geographic overview of macro-regions (boxed numbers) and their corresponding key sites (small numbers); 1 = Southern Scandinavia (SS), 2 = Lithuania (LT), 3 = Northern Germany (NG), 4 = Britain (GBS), 5 = Poland (PL), 6 = Belgium and Southern Netherlands (BSN), 7 = Bohemia and Moravia (BOMO), 8 = Southern Germany (SG), 9 = Northern France (NF), 10 = Austria, Slovakia and Hungary (ASH), 11 = Switzerland (CH), 12 = Northeastern Italy (NEI), 13 = Western France (SWF), 14 = Cantabrian Spain (IBC), 15 = Mediterranean Iberia (IBM), 16 = Atlantic Iberia (IBA). (**b**) Time slices I to IV in relation to established chronological schemes in Late Glacial/incipient Holocene archaeology. (**c**) Number of expert-submitted named archaeological cultures (NACs) per time slice and macro-region.

The deep prehistory of European forager populations is commonly framed in cultural-historical terms^1,2,5,34–36^ – that is, as a sequence of variously overlapping taxonomic units, although the precise definition of these units as ‘techno-complexes’, ‘techno-cultures’ or ‘industries’ may differ between research contexts^24,37^. Taxonomic units or ‘named archaeological cultures’ (NACs) are the most widely recognized higher-order archaeological units used to interrogate the long-term evolution of Pleistocene societies. NACs thus offer a suitable higher-order reference system for organizing and synthesizing currently available knowledge on lithic behavioral variability and evolution across different parts of Late Glacial Europe. NACs are situated in space and time: we employ macro-regions reflecting geographic and ecological differences but also scholarly expertise and predefined time slices (binned in equidistant 1k-year intervals) to construct the data framework. Geographic macro-regions (n = 16) and employed time slices (n = 4) are shown in Fig. 1 (cf. Supplementary Table 1). Time slices I to IV span the period between 15 and 11 ka BP, including the terminal part of the Pleniglacial (Late Upper Paleolithic), the Late Glacial (Final Paleolithic) and the incipient Holocene (earliest Mesolithic). The individual NACs (n = 86) were assembled from the literature based on the current state-of-the-art of lithic research in a given macro-region and then assigned to a single or multiple time slices depending on the latest relative, stratigraphic and/or chronometric dating evidence. For each NAC, a broad range of lithic variables pertaining to different levels of technological organization were then extracted from the currently available literature by expert editors acquainted with a given macro-region.

### Collaborative data compilation and recording process

**Fig. 2:**
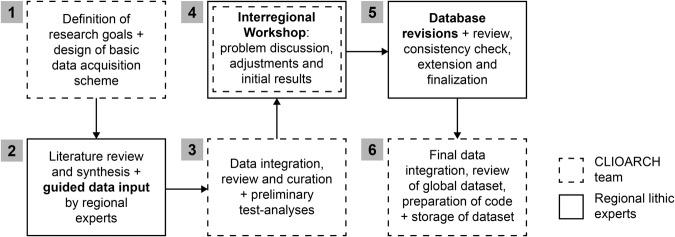
Workflow of collaborative data compilation and synthesis. Numbers indicate successive stages of data processing, from definition and preparation to validation.

**Fig. 3:**
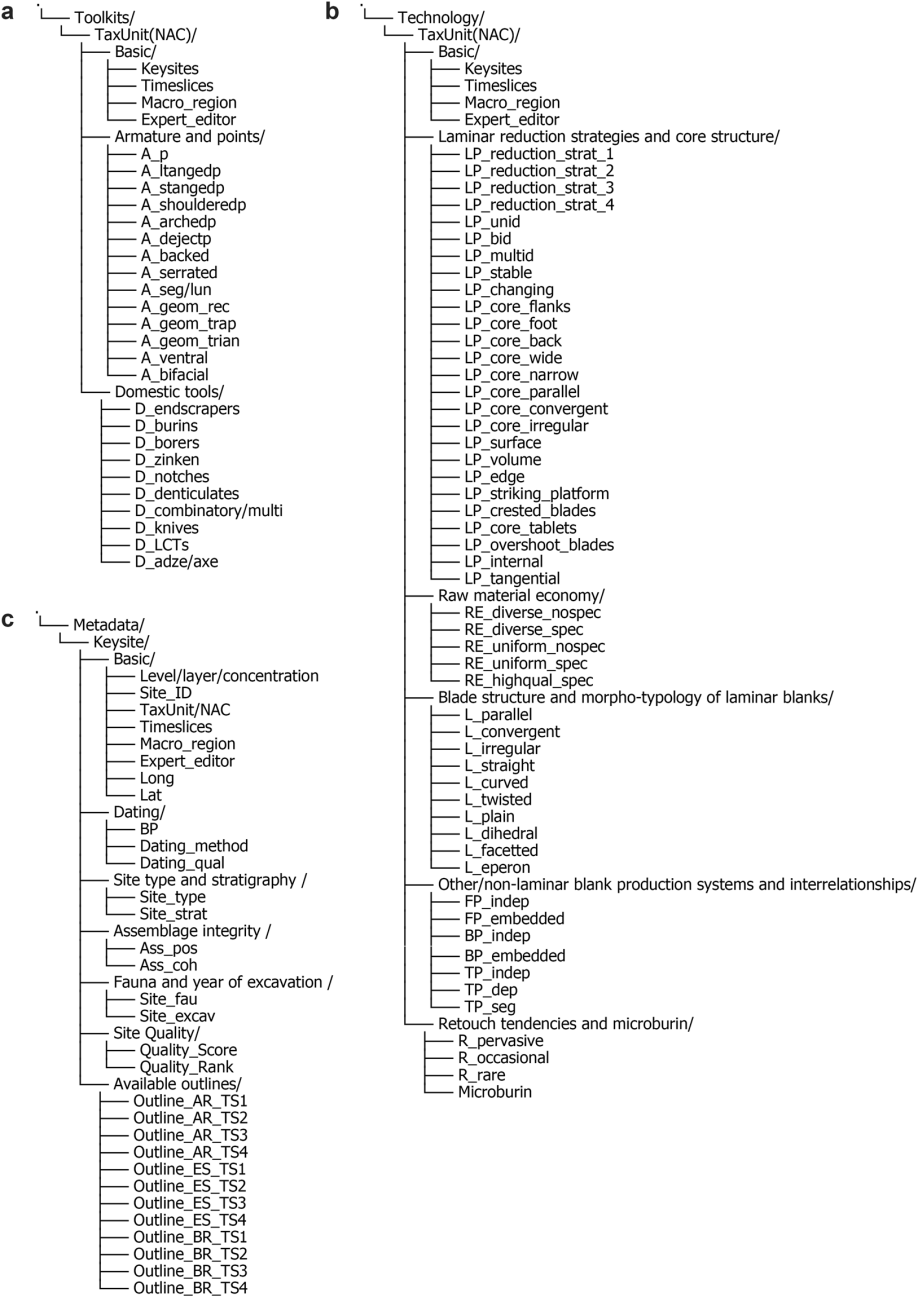
Trait structure of main data domains and site-specific metadata. (**a**) Toolkits data domain (I). (**b**) Technology data domain (II). (**c**) Metadata.

The workflow of collaborative data compilation, preparation and synthesis is outlined in Fig. 2. Each macro-region was handled by an expert editor intimately familiar with the regional lithic evidence and the latest archaeological debates and interpretations. In a first step, regional lithic experts carefully reviewed the literature and identified the latest culture-historical framework accepted for a given macro-region, thereby assembling a regional inventory of NACs. Expert editors were then confronted with a first draft recording scheme integrating a broad range of lithic variables and asked to synthesize and test-record the published lithic data for each inventoried NAC within their macro-region accordingly. Initial insights and encountered problems were subsequently discussed by all research participants on a two-day online workshop to adjust and improve the data infrastructure and to align expectations and basic definitions as well as to address open questions^21^. Based on the critical feedback received, the lithic data structure was updated, and all initial entries thoroughly checked by the CLIOARCH team. Datasets were then sent back to the expert editors, who had to review and revise their provided data and address any identified issues and inconsistencies. Expert editors were supported by CLIOARCH core members throughout the entire process to ensure overall data coherence and to provide feedback and/or a second opinion if needed. The revised macro-regional datasets were then submitted for final data integration and preparation. The recorded individual lithic variables are shown in Fig. 3 and described in detail in Tables 2–9 (see below) and in Data Records.

### Dataset design and data collection principles

The 1511NAC dataset^19^ is designed to facilitate macro-archaeological analyses of lithic diversity, complexity and cultural evolution in Europe between 15 and 11 ka BP^21^. Part of this endeavor is the examination of potential offsets between different lithic data domains and/or dimensions of lithic production technologies and toolkits. The overall structure of the dataset^19^ reflects this analytical aim (Table 1): the first data domain (I) gathers NAC-level information on the structural composition of lithic toolkits based on a broad distinction of lithic morphotypes divided into ‘armatures’ and ‘domestic’ tools respectively (Tables 2, 3); the second data domain (II) draws together NAC-level lithic information on the organization of production technologies in the form of discrete traits, focusing especially on the laminar component (Tables 4–9); the third data domain (III), finally, consists of a library of 2D outline shapes of key tool groups (armatures, endscrapers and borers/perforators) extracted from the literature and digitally prepared for analysis with site-level resolution. The first and second data domains are fed with presence/absence data (categorical) coded as binary characters (1/0), or as n/a in cases where the respective information was not available or ambiguous. Cases for which a decision between 1 or 0 could not reasonably be made based on the present state of publication were originally recorded as ‘?’, but have been amended to ‘n/a’ as the latter is more conservative and inter-observer disparities are otherwise likely to introduce yet another artificial layer of variability. Presence data coded as 1 represents NAC-wide recurrence of occurrence, and thus records technological traits and tool morphotypes that are consistently but not necessarily always observed within archaeological sites grouped under a given regional NAC. The third data domain consists of digitized shape data (continuous) from individual key sites attributed to regional NACs, provided as prepared image files and outlines. The contents of all data domains are described in more detail in the next section.

**Table 1:** Structure of the dataset^19^ and descriptive parameters of the main data domains.

Data domains	Sub-domains	Number of recorded traits	Data type	Total observations	Data resolution
*Toolkit*	Armature/points; Domestic tools	24	Discrete	86	NAC-level
*Technology*	Laminar reduction strategies and core structure; Raw material economy; Blade structure and morpho-typology of laminar blanks; Other/non-laminar blank production systems and their interrelationships; Retouch tendencies ( + microburin)	52	Discrete	86	NAC-level
*2D Artefact outlines*	Armature (AR); Endscraper (ES); Borer (BR)	34 (AR)	Continuous	5986 (AR = 3512, ES = 1930, BR = 544)	Object-level (and site-level)
Metadata	Site context and data quality; Calculated site-quality scores; Register of processed and recorded tool outlines	25	Various	—	Site-level

**Table 2:** Categorical NAC-level lithic armature data recorded as systematic presences/absences in data domain I (Toolkits).

Code	Description
A_p	Simple points
A_ltangedp	Large tanged points (width >15 mm)
A_stangedp	Small tanged points (width <15 mm)
A_shoulderedp	Shouldered points
A_archedp	Arched/Arch-backed points
A_dejectp	Dejected/Angle-backed points
A_backed	(Other) Backed pieces
A_serrated	Serrated/denticulated implements
A_seg/lun	Segments/lunates
A_geom_rec	Rectangular (geometric) microliths
A_geom_trap	Trapezoid (geometric) microliths
A_geom_trian	Triangular (geometric) microliths
A_ventral	Partial ventral retouch
A_bifacial	Complete bifacial retouch (shaping)

**Table 3:** Categorical NAC-level domestic lithic tool data recorded as systematic presences/absences in data domain I (Toolkits).

Code	Description
D_endscrapers	Endscrapers
D_burins	Burins
D_borers	Borers/perforators (including *becs* and other atypical forms)
D_zinken	*Zinken*
D_notches	Notched pieces
D_denticulates	Denticulated pieces
D_combinatory/multi	Combinatory tools/multitools
D_knives	Large blade knives/heavily retouched larger blades
D_LCTs	(Other) Large Cutting Tools
D_adze/axe	Flake adzes/axes

**Table 4:** Categorical NAC-level data on laminar reduction strategies, core structure and exploitation surface geometry recorded as systematic presences/absences in data domain II (Technology).

Code	Description
LP_reduction_strat_1	One single focal reduction strategy
LP_reduction_strat_2	Two focal reduction strategies
LP_reduction_strat_3	Three focal reduction strategies
LP_reduction_strat_4	Four focal reduction strategies
LP_unid	Undirectional laminar production
LP_bid	Bidirectional laminar production
LP_multid	Multidirectional laminar production
LP_stable	Reduction patters are stable across core life-histories
LP_changing	Reduction patterns change across core life-histories
LP_core_flanks	Prepared/well-defined core-flanks
LP_core_foot	Prepared/defined core foot
LP_core_back	Prepared/defined core back
LP_core_wide	Wide reduction surfaces
LP_core_narrow	Narrow reduction surfaces
LP_core_parallel	Geometry of reduction surfaces highlights parallel configurations
LP_core_convergent	Geometry of reduction surfaces highlights convergent configurations
LP_core_irregular	Irregular reduction surface geometries

**Table 5:** Categorical NAC-level data on core volume management and preparation recorded as systematic presences/absences in data domain II (Technology).

LP_surface	Surface exploitation of cores (surface-near volumes are targeted for exploitation)
LP_volume	Volume(tric) exploitation of cores (core reduction concepts in principle allow for the exploitation of whole core volumes)
LP_edge	(Narrow) Edge exploitation (production of blanks from natural or artificial narrow edges without exploitation of whole core volumes, including burin-like productions)
LP_striking_platform	Prepared striking platform of cores
LP_crested_blades	Crested/neocrested blades
LP_core_tablets	Core tablets
LP_overshoot_blades	Preparatory/corrective overshot blades
LP_internal	Internal knapping (knapping gesture results in point of impact on the interior of the striking platform, often associated with thick blank platforms/butts and pronounced striking features such as bulbs)
LP_tangential	Near-edge/peripheral knapping (knapping gesture results in point of impact very close or at the core edge, often resulting in marginal blank platforms and diffuse striking features)

**Table 6:** Categorical NAC-level raw material economy data recorded as systematic presences/absences in data domain II (Technology).

Code	Description
RE_diverse_nospec	Focus on a diverse raw material base but no obvious specialization (i.e., no specific link between a particular raw material and a particular reduction strategy)
RE_diverse_spec	Focus on a diverse raw material base with evidence on specialization on (a) selected raw material(s)
RE_uniform_nospec	Focus on uniform raw material base yet no obvious specialization, i.e., adaptation of the knapping strategy to the given raw material (properties, forms)
RE_uniform_spec	Focus on a uniform raw material base with evidence for specialization, i.e., adaptation of knapping strategies to raw material conditions/properties
RE_highqual_spec	Global focus on high quality raw material with evident adaptation of knapping strategies to the properties of the raw material

**Table 7:** Categorical NAC-level data on blade structure and the morpho-typology of laminar blanks recorded as systematic presences/absences in data domain II (Technology).

Code	Description
L_parallel	Parallel outlines
L_convergent	Convergent outlines
L_irregular	Irregular outlines
L_straight	Straight laminar profiles
L_curved	Curved laminar profiles
L_twisted	Twisted laminar profiles
L_plain	Plain platforms
L_dihedral	Dihedral platforms
L_facetted	Facetted platforms
L_eperon	*En éperon* platform preparation

**Table 8:** Categorical NAC-level data on other (non-laminar) reduction systems and system-interrelationships recorded as systematic presences/absences in data domain II (Technology).

Code	Description
FP_indep	Independent flake production
FP_embedded	Embedded flake production (dependent on laminar production(s))
BP_indep	Independent bladelet production
BP_embedded	Embedded bladelet production (dependent on blade or flake production(s))
TP_indep	Tool production/manufacture independent of blank production (i.e., no systematic link between specific blank and tool forms/types)
TP_dep	Tool production/manufacture dependent on or follows/ anticipates blank production (i.e., evidence for a systematic link between specific blank and tool forms/types)
TP_seg	Segmentation of domestic tools and armature (blank-tool relations differ between the two, different blanks are selected and transformed in both cases, either within the same reduction strategy or the two tool categories are produced from separate reduction strategies)

**Table 9:** Categorical NAC-level data on retouch-tendency and microburin technology (all lithic artefacts ≥2 cm) recorded as systematic presences/absences in data domain II (Technology).

Code	Description
R_pervasive	Assemblage-level retouch is pervasive (>20%)
R_occasional	Assemblage-level retouch is occasional (10–20%)
R_rare	Assemblage-level retouch is rare (<10%)
Microburin	Microburin products and by-products

## Data Records

### Folder structure

**Fig. 4:**
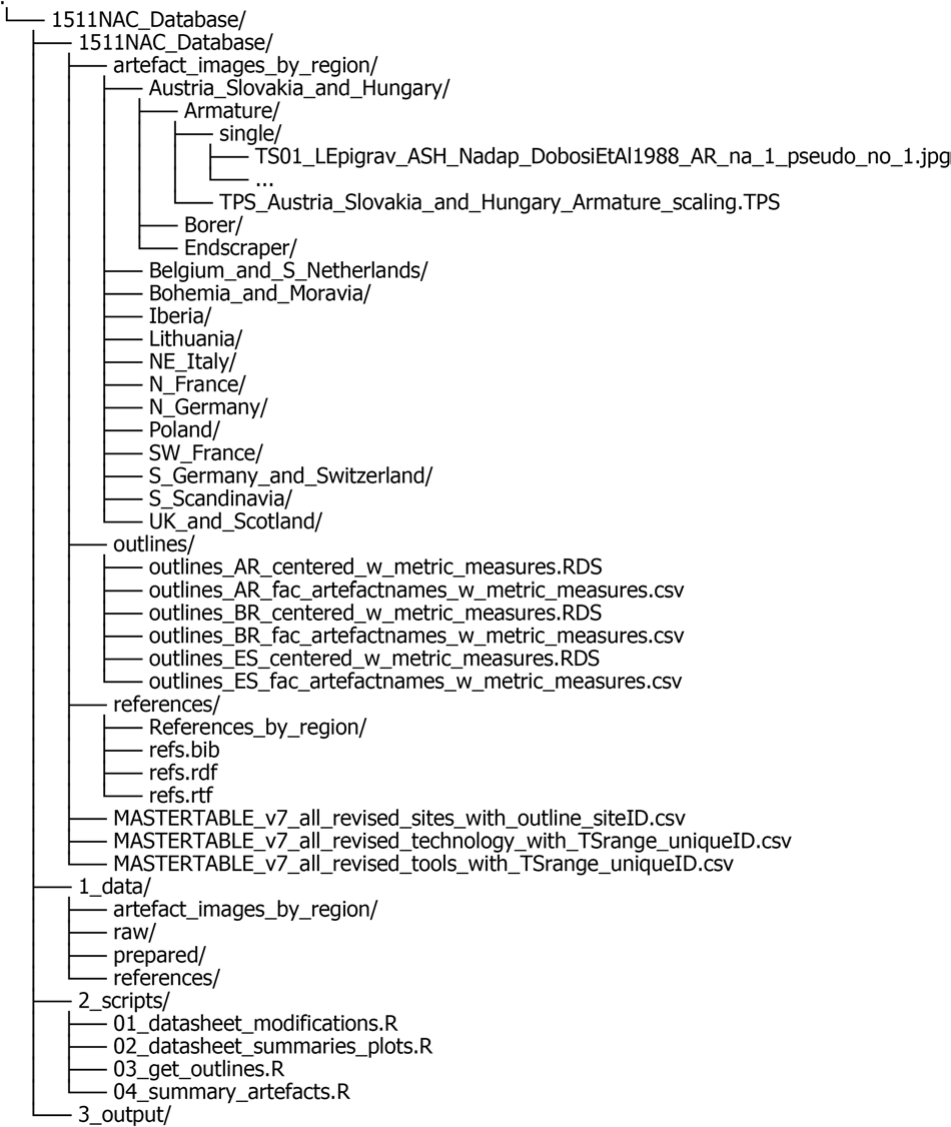
Folder tree structure of the data repository^19^.

The complete 1511NAC dataset is openly available at Zenodo^19^ and structured as shown in Fig. 4. The data repository comprises four principal folders: *1511NAC_Dataset*, *1_data*, *2_scripts* and *3_output*. Generally speaking, the folder *1511NAC_Dataset* contains all data entries relevant for researchers who are interested in using the dataset^19^. The folders *1_data*, *2_scripts* and *3_output* contain the raw data and R scripts that were created and used to generate the dataset^19^ and the figures in this paper. Each principal folder is described in more detail below.

*1511NAC_Dataset*

- The *artefact_images_by_region* subfolder holds the prepared images of all single lithic artefacts in .jpg format, organized per region and ordered according to the three broader artefact groupings (data domain III/2D artefact outlines, see below). Each .TPS file (thin-plate-spline) contains the pixel-to-centimeter ratio information for each set of lithic artefacts per region per artefact grouping, enabling further metric analysis.
- The *outlines* subfolder contains all .RDS 2D outlines generated from the prepared artefact images. For each of the three larger artefact groupings (armature, endscrapers, borers/perforators, see below), a single .RDS file containing the respective artefact outlines with their metadata and metric measurements is provided, as well as a separate .csv file holding their respective metadata and metric measurements alone, and thus facilitate simple metric analyses. To display the .RDS files properly in R, the *Momocs* package (≥1.4.0^38^) must be installed and loaded.
- The *references* subfolder gathers all primary literature referenced in the dataset^19^ prepared in different file types to be directly imported into currently available bibliographic management tools.
- The three master tables are provided as separate .csv files. They contain the metadata of all archaeological key sites represented in this dataset^19^ (*MASTERTABLE_v7_all_revised_sites_with_outline_siteID.csv*) as well as the recorded trait data of data domains I and II (Toolkits: *MASTERTABLE_v7_all_revised_tools_with_Tsrange_uniqueID.csv*; and Technology: *MASTERTABLE_v7_all_revised_technology_with_TSrange_uniqueID.csv*).

*1_data*

This folder contains both the raw and the final data as contained in the 1511NAC_Dataset, as well as the data required to reproduce the figures and tables included in this paper. The files and the dataset^19^ included here are primarily provided for full transparency. They allow researchers to trace the entire process of data processing leading to the final dataset^19^ supplied.

- The *artefact_images_by_region* subfolder is identical as the above-described.
- The *raw* subfolder contains the original, unprocessed .csv files holding the site-specific metadata as well as the trait-data of data domains I and II as submitted by the macro-regional expert editors.
- The *prepared* subfolder contains all data derived from the raw master tables and the prepared lithic artefact images, etc., as well as all transformed data. The folder therefore contains all data necessary to reproduce the figures and tables in this paper and includes the same final outline data and master tables as contained in the main *1511NAC_Dataset* folder.
- The *references* subfolder is identical as the above-described.

*2_scripts*

This folder exists purely for the purpose of reproducibility and contains the R scripts used to prepare and extract the data which are then deposited in *1511NAC_Dataset*, but also the scripts used to generate the figures and tables of this paper.

- *01_datasheet_modifications.R* is used to prepare the spreadsheets containing information on archaeological key sites and those holding data domains I and II.
- *02_datasheet_summaries_plots.R* is used to data-wrangle and to create the figures and tables in this paper related to site-specific metadata and data domains I and II.
- *03_get_outlines.R* is used to extract outlines from the supplied .jpg images, to further prepare them, and to calculate their associated metric measurements.
- *04_summary_artefacts.R* is used to data-wrangle and to create the 2D outline-related figures and tables shown in this paper.

*3_output*

This folder contains the original figures and tables created for this article.

Further information and explanation on file naming conventions in the dataset^19^ can be found in the Supplementary Information.

### Analytical units and data domains in detail

The general trait-structure of the dataset^19^ is shown in Fig. 3. The primary analytical units are the macro-regional NACs and in the case of outline data, both NACs and individual archaeological key sites. We provide each data domain as a separate .csv file for easy and flexible implementation. Linked metadata provides further contextual information on the key sites respectively attributed to macro-regional NACs, such as lithic assemblage provenance, site quality, radiometric dating, and the underlying bibliographic sources.

#### Cultural taxonomic units

The dataset^19^ comprises 86 separate NAC entries. Supplementary Table 3 shows how these are spread over time slices I to IV and the 16 captured macro-regions. These NACs cover a large portion of Northern and Western Europe and circumscribe the following geographic macro-regions: Atlantic Iberia, Cantabrian Iberia, Mediterranean Iberia, Western France, Britain, Northern France, the Low Countries, Switzerland, Southern Germany, Northern Germany, Southern Scandinavia, Northeastern Italy, Austria/Slovakia/Hungary, Moravia/Bohemia, Poland, and Lithuania. NAC naming conventions follow common literature practice, but priority was always given to the state-of-the-art understanding of regional experts. NACs were either recorded as bounded units (e.g., ‘Magdalenian’, ‘Federmesser’, ‘Ahrensburgian’ or ‘Belloisian’) or as chronological sub-units (e.g., ‘Final Magdalenian’, ‘Havelte’, ‘Early Azilian’ or ‘Late Laborian’), depending on the importance of the latter in the archaeological discourse and the prevailing opinion/consensus in the respective body of expert literature. Individual NACs identified and recorded in this way are linked to a portfolio of key archaeological sites anchoring them in space and time and exemplifying the lithic information assembled in the toolkit and technology data domains (n = 350). These sites tend to be the best-studied site exemplars of a NAC in each macro-region, hold key information on the chronological position of said NAC, and/or frame a rich source of drawings or photographs of NAC-specific lithic tools.

#### Toolkit data

Discrete binary toolkit data (presence/absence) for different NACs is provided for 24 tool morphotypes subdivided into two broader groups of ‘armatures’ and ‘domestic’ tools, constituting data domain I. The armatures group mainly gathers various lithic point types, backed implements and microlithic forms, while the domestic tools group covers the near-ubiquitous Upper Paleolithic tool types of burins, endscrapers and borers/perforators. The following morphotypes are included in the armatures group (n = 14): simple points, large tanged points (width >15 mm), small tanged points (width <15 mm), shouldered points, arched/arch-backed points, dejected/angle-backed points, (other) backed pieces, serrated/denticulated implements, segments/lunates, rectangular (geometric) microliths, trapezoid (geometric) microliths and triangular (geometric) microliths, as well as separate entries for the recurrence of NAC-level partial ventral and bifacial retouch (cf. Table 2). The domestic tools group sports the following morphotypes (n = 10): endscrapers, burins, borers (including perforators, *becs* and other atypical forms), *Zinken* (borers with a typical, strongly bent nose), notched pieces, denticulated pieces, combinatory tools/multitools, large blade knives/heavily retouched larger blades, (other) LCTs (Large Cutting Tools) and flake adzes/axes (cf. Table 3). Presence data (1) indicates NAC-level recurrence and thus inter-assemblage importance of these morphotypes.

#### Technology data

Discrete binary data on technology (presence/absence) for different NACs is recorded in 54 individual techno-typological traits, organized in five sub-domains which together make up data domain II. Again, these data operate on the aggregate level and the recorded information refers to NAC-wide trends and patterns not necessarily reducible to observations pertaining to individual archaeological sites. We assume that NAC-level records represent the observations made on multiple archaeological key sites and so provide better information overall. Building on a long tradition of lithic technological analysis, the five technology sub-domains are: (A) laminar reduction strategies and core structure, (B) raw material economy, (C) blade structure and morpho-typology of laminar blanks, (D) other blank production systems and system interrelationships, (E) retouch tendencies and microburins. Sub-domain A encompasses 26 traits pertaining to the number of co-existing laminar core reduction strategies (distinct modes of working selected lithic raw material nodules), the extent and dynamics of core preparation (e.g., investment and stability of approach), the geometry of reduction surfaces (shape and edge configuration), and the knapping technique (internal vs. tangential) (cf. Tables 4, 5). Sub-domain B draws together 5 traits related to the relationship between raw material choice and reduction strategies (cf. Table 6). Sub-domain C features 10 traits linked to the morphology, profile and platform configuration of laminar blanks (cf. Table 7). Sub-domain D gathers 7 traits targeting the interdependency of different blank production systems (flake, blade, bladelet) and the relationship between blank production and tool manufacturing (cf. Table 8). Sub-domain E, finally, compiles 4 traits pertaining to the scope/intensity of recurrent assemblage-wide retouch (frequency of tools/modified pieces) and as the microburin technique (cf. Table 9). These discrete trait data enable the examination of relationships between sub-domains and broader spatiotemporal trait dynamics.

#### 2D artefact outlines

**Fig. 5:**
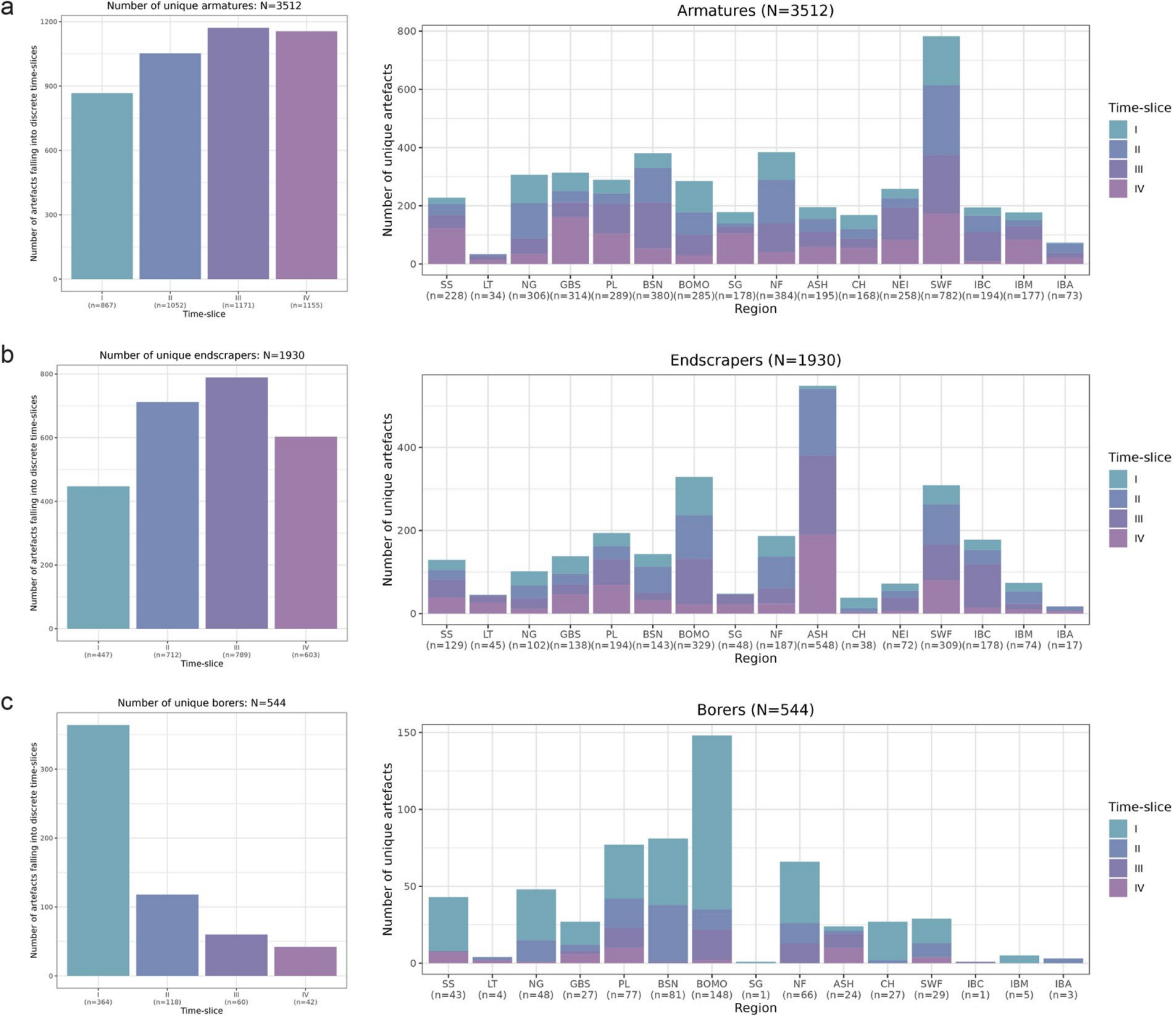
Overview of lithic artefact outlines in the dataset^19^. (**a**) Number of digitized armature outlines (AR) for each time slice (*left*) and their distribution across individual macro-regions (*right*). (**b**) Number of digitized endscraper outlines (ES) for each time slice (*left*) and their distribution across individual macro-regions (*right*). (**c**) Number of digitized borer/perforator outlines (BR) for each time slice (*left*) and their distribution across individual macro-regions (*right*). Note that the borer dataset includes incomplete artefacts.

The 2D outline library (data domain III) hosts 5986 individual lithic tool outlines, compiled and grouped in three broader categories: armatures (AR), endscrapers (ES), and borers (BR) with reference to the categories listed in the toolkit data domain. Burins were not collected because of the huge variability of artefact shapes associated with this tool group and because the so captured variability is shaped by extreme levels of blank-derived noise. The AR group comprises 3512 artefact outlines linked to tool morphotypes classified as ‘armatures’. The ES group contains 1930 outlines identified as ‘endscrapers’. The BR group contains 544 outlines identified as ‘borers’ and perforators. AR and ES outlines were collected from complete lithic artefacts only, or artefacts for which the missing part (for example, the apex of a point) could unambiguously be reconstructed. BR outlines do not respect this criterion and must therefore be handled with caution; here, focus rests on the working end of the artefacts rather than the total outline. These data are provided both as fully processed outlines saved as .RDS in R to preserve as much information as possible, and as individual image files pre-processed into binary artefact images which can directly be fed into outline extraction and shape-description workflows^39^. These data can thus be deployed in outline analyses, using geometric morphometric methods of shape space analysis^40^. They provide the hitherto largest repository of key tool forms from the European Late Glacial. Each artefact image is labelled according to the conventions defined in the Supplementary Information (Part 4, cf. Supplementary Table 8), specifying its NAC, time slice, macro-region, site of origin, and bibliographic reference(s). In addition, AR outlines come with supplementary information on point axiality, distinguishing between distal (‘d’) and proximal (‘p’) lithic points if possible. A breakdown of the spread of outlines across time slices and macro-regions is provided in Fig. 5.

The outline datasets^19^ also harbors metric artefact-level information, even though this data is only available at the sub-sample level and was not systematically collected. Where scale bars were available on the submitted images (AR: n = 2966; ES: n = 1628; and BR: n = 498), a pixel-to-centimeter ratio/scaling factor was created for each image separately using *tpsUtil* and *tpsDig2* in R^41^. This scaling factor was then implemented in the outline extraction process^39^ using the *outlineR* package^42^ to automatically associate a scaling factor – where applicable – with individual outlines, allowing for the quick and ready extraction of a multitude of user-defined artefact-specific metric measurements. The dataset^19^ supplies the following artefact-level measurements extracted via the *Momocs* package^38^: length (cm), width (cm), area (cm^2^), perimeter (cm), calliper (cm), circularity and eccentricity (see^38^ for a definition and description of these specific measurements).

#### Metadata

Contextual and supplementary information on the selected archaeological key sites (Supplementary Tables 2, 5) anchoring regional NACs in the dataset^19^ is organized according to three sub-domains. The first harbors information on site location (geographic coordinates), dating range, dating method, dating quality, the type of site, the nature, context, and coherence of the lithic assemblage(s) in question, faunal preservation as well as the original year of excavation (Table 10). Sub-domain two addresses the quality and reliability of the key sites listed by calculating quality scores (1 to 6) and ranks (1 to 4) based on information provided in the first sub-domain (Supplementary Tables 4, 6). Lower quality scores broadly indicate less reliable archaeological contexts expressed in lower ranks. The third and last sub-domain is a register of processed tool outlines stored in the outline library so that analysts can quickly identify the distribution of available outlines across sites (Supplementary Table 7).

**Table 10:** Basic site-specific information and data quality indicators.

Code	Description
Long	Longitude coordinates (given in decimal degree)
Lat	Latitude coordinates (given in decimal degree)
BP	Broad dating range in cal. BP, e.g., “15-13 calBP”
Dating_method	Dating method, e.g., “14 C”, “TL”, “typology”, “technology”, “biostratigraphy” or “geology”
Dating_qual	Dating quality (drop-down): “Reliable” or “Problematic”
Site_type	Type of site (drop-down): “Openair”, “Cave” or “Rockshelter”
Site_strat	Stratification (or not) of site (drop-down): “Stratified” or “Surface”
Ass_pos	Assemblage position (drop-down): “Primary/insitu” or “Secondary/relocated”
Ass_coh	Assemblage coherence (drop-down): “Homogeneous” or “Mixed”
Site_fau	Presence/preservation of faunal material (drop-down): “Preserved” or “No”
Site_excav	Period of excavation (drop-down): “Before WW2”, “1950–1980”, “1980–2000” or “After 2000”

#### Dataset coding and abbreviations used

Metadata (NACs, macro-regions, expert editors, etc.), tool groups, technological traits, 2D artefact outlines and archaeological site contexts are coded as abbreviations. All abbreviations used in the dataset^19^ are listed in the foregoing tables or are explained in the Supplementary Information.

## Technical Validation

### Data quality and reliability

**Fig. 6:**
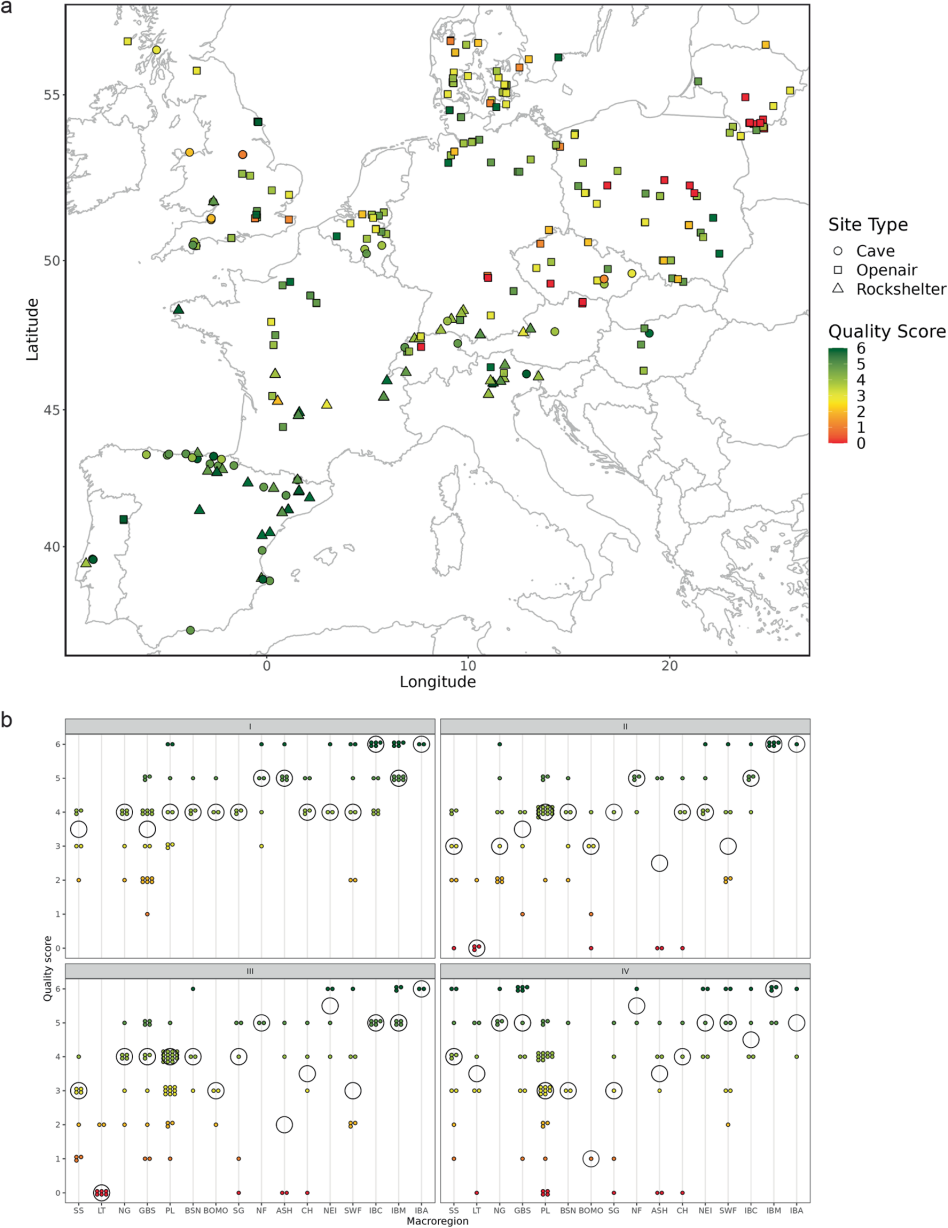
Data quality. (**a**) Quality scores computed for each archaeological key site (see text for explanation) from 0 = poor to 6 = excellent. (**b**) Detailed break-down of site-specific quality scores in relation to time slices and individual macro-regions (Supplementary Table 4). Black circles indicate median values computed for each macro-region within a given time slice.

Quality scores were calculated for each archaeological site context based on the associated metadata provided (Supplementary Table 4). The score was derived by summing all positive entries (1s) for the following data categories: dating quality = reliable, site stratigraphy = stratified, assemblage position = primary/*in-situ*, assemblage coherence = homogeneous, and excavation year = 1980–2000 (Supplementary Table 6). Recorded years of excavation = after 2000 (1s) were double weighted, resulting in a possible score of 6 for the most dependable archaeological contexts. Figure 6a shows the geographic distribution of quality scores across Europe, pointing to north-south and east-west quality gradients with Western and Southern European sites scoring generally higher. This pattern is mainly a function of the availability and inclusion of open-air vs. sheltered sites (caves, rockshelters) in the dataset^19^. In addition, it may highlight differential depositional and preservation conditions of stone artefact assemblages on the North European Plain and in high-latitude landscapes, which have been strongly affected by glacial activity, for example across the northeastern extent of the study area. Some of the included lithic data from Eastern and parts of Central Europe may thus be less reliable than their Southern and Western European counterparts and this is in part also a consequence of the varying scope and intensity of Late Glacial research in these regions in recent decades. This situation is also well-reflected in the distribution of average quality scores across macro-regions plotted in Fig. 6b. Lithuania and the macro-region comprised of Austria, Slovakia and Hungary host a notable number of comparatively problematic data contexts and should accordingly be treated with some caution. Interestingly, the average quality scores of all included macro-regions, taken together, slightly deteriorate over time (from TS I to IV), and not the other way around as may be expected *a priori*. This similarly suggests that research history and/or unequal levels of sedimentation may play a role here, although past human behavior may also be implicated, for example due to more mobile forager groups and hence possibly more elusive material signatures in some parts of Northern and Eastern Europe. Note that this quality assessment does not take into consideration how the lithic data was analyzed and formed, for example whether or not the latest methods and analytical standards were employed.

## Usage Notes

### Data structure and spatiotemporal scale

**Fig. 7:**
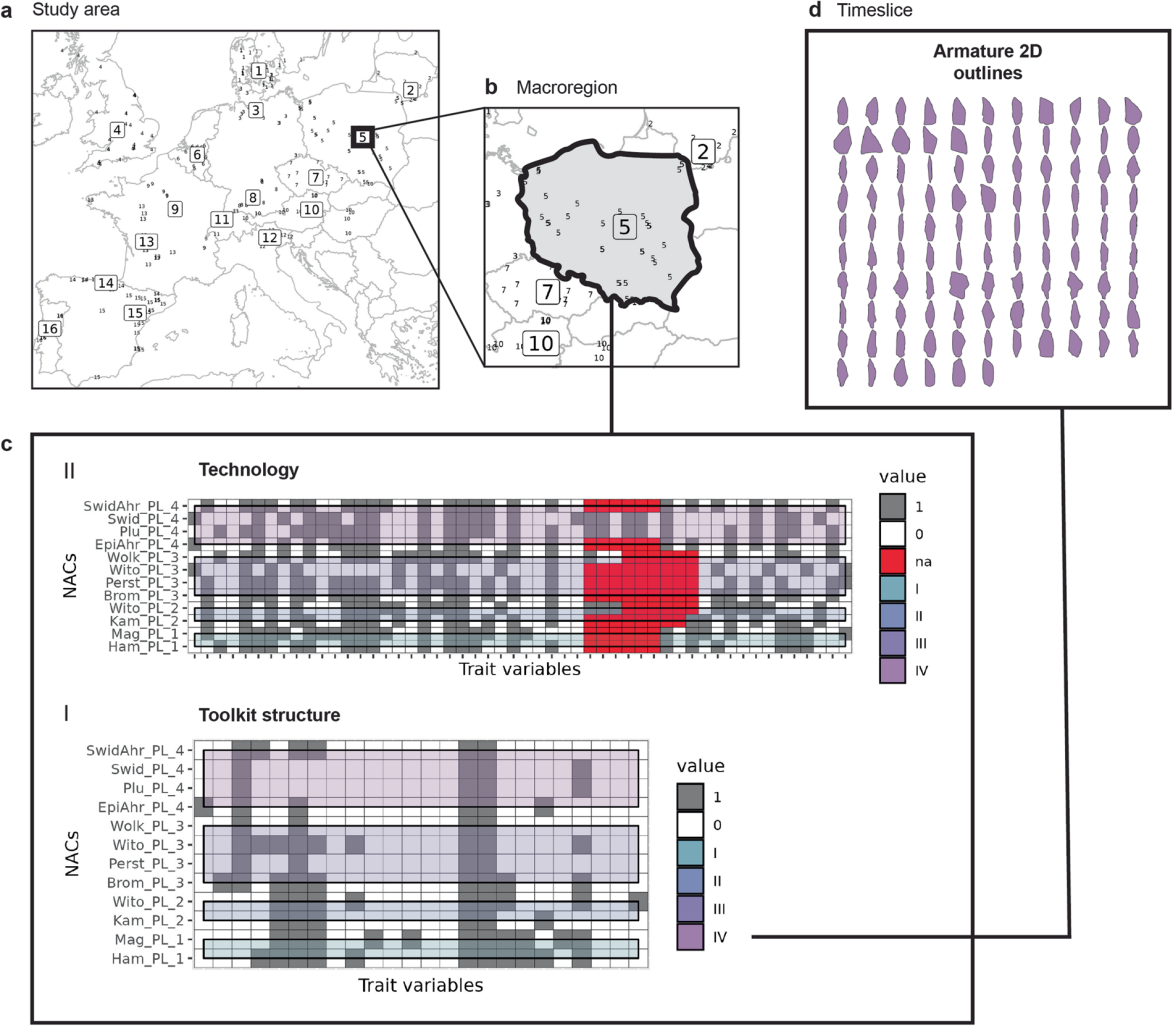
Overview and exemplification of lithic data structure. (**a**) Pan-European scale of observation with all 16 macro-regions (boxed numbers) and their associated archaeological key sites (small numbers). (**b**) Macro-regional scale of observation (example: Poland); (**c**) Macro-regional data matrix of observations on toolkit composition (*I*) and technology (*II*) coded as presences/absences (na = information not available in the literature) and sorted according to time slices. (**d**) Digitized 2D outlines of lithic armature within a selected time slice (example: TS IV) of the same macro-region. This data structure facilitates the diachronic and synchronic analysis of within and cross-domain lithic variation.

The dataset^19^ records multi-scale observations on lithic technology for each macro-region (Fig. 7). Data on reduction technologies and toolkit composition are provided as NAC-level presence/absence matrices (macro-regional scale) but the resolution of the two data domains differs, as toolkit trait variables refer to highly generalized lithic tool morphotypes while technological traits represent a mix of well-established descriptive variables and more synthetic observations. Variability in the technology domain is thus not directly comparable to variability in the toolkit domain, but we generally expect that technology-toolkit relationships reflect relevant information on technical choices and cultural evolution. In other words, patterns in macro-regional technological data should be related to the structural organization of the corresponding toolkits, but the relationship must not always be strictly co-evolutionary as technical trade-offs may for example promote informal tool-use not documented in the dataset^43^. Conversely, tools shapes may be more readily copied and tool concepts may thus be more mobile between communities (reflected in *Tools*-based similarity and co-occurrence of rarer forms such as *Zinken*, specific geometric microliths or tanged points), whereas specific ways of working lithic raw material volumes (combinations of *Technology* variables) are often expected to diffuse less easily^44,45^, but this hypothesis should be empirically explored and different techno-cultural contexts may yield varying dynamics^46,47^. The offered data structure so enables the comparative investigation of patterned co-variation between technology and toolkit data, and to test whether such relationships differ between regions and time slices. NAC-level data resolved on the macro-regional scale (Fig. 7a,b) can thus generally be examined diachronically (analytical units: different time slices) or synchronically (analytical units: same time slices across regions).

The outline data adds depth to this structure. On the temporal axis, the artefact outlines are also resolved according to the four time slices, while spatially they are linked to individual archaeological key sites and thus enable the investigation of geographically continuous dynamics of shape change in three different artefact registers (armatures, endscrapers and borers). Again, the relationship between patterns recorded in artefact outline assemblages and NAC-level toolkits should not be arbitrary, but the nature of the interactions is open to characterization. Depending on the spatiotemporal, and possibly technological, context, diversity in armature morphotypes (*Tools*) may be directly linked to global diversity patterns in armature outlines (*Outlines*), or not. This is because *Outline* data should capture more subtle levels of variation but also since the variability within morphotypes (as defined in *Tools*) may vary between technological contexts. Diversity in tool outlines may similarly be expected to be elevated in cases where lithic technology is rooted in a conceptual and operational distinction between lithic blank and tool production, so that tool confection (modification) becomes the key means to install and differentiate tool functions, or, alternatively, reflect the grounding diversity of forms obtained during blank production, or a combination of both. These and cognate hypotheses can empirically be tested using the present dataset^19^, and changing structuration principles at the intersection of technology, toolkit structure and artefact shape may in this way be discovered and pinpointed in time and space.

### Inter-domain dynamics

The multi-domain and multi-scale structure of the dataset^19^ supports the systemic analysis of long-term dynamics of lithic change. By quantifying the amplitude and rate of change in varying data domains, researchers can examine patterns of synchronicity or lack thereof in the evolution of stone artefact technologies on a pan-European scale, and in this way contribute to debates on the temporality of material culture change^48–52^ as well as broader discussions on the evolution of cultural systems^53,54^. Confronting the dynamics of change across data domains thereby contributes to the identification, quantification, and hence qualification of different tempi and modes of change, possibly revealing that technological choices, toolkit compositions, and artefact shapes are subjected to differential dynamics of long-term technological evolution. Under which conditions such asynchronies in lithic evolution emerged, or whether we should instead regard such patterns as the default mode of material culture change, is an important emerging question and the dataset^19^ makes it possible to begin systematically addressing it. Inter-domain analysis and comparison finally provide a new means to assess the general character and the complexity of cultural evolution between ~15,000 and 11,000 years ago. Complexity can for example be understood as a property of domain interaction, and mapping out domain-specific dynamics of change can thus provide new perspectives on questions of linearity and nonlinearity in the millennial-scale evolution of Late Pleistocene/earliest Holocene human forager technologies.

### Further remarks and perspectives of data usage

Archaeological data are voluminous, distributed across many language and publication formats. They are rarely available in standardized digital formats. Mindful of the analytical decisions that had to be taken in transforming qualitative observations and legacy images into the dataset^19^ presented here^55,56^, we recommend due diligence in its usage. That said, computational tools are offering new avenues for research within lithic analysis specifically^57,58^, and for understanding large-scale patterns of cultural evolution^59^. Data availability is a key element in this endeavor^60,61^ and the dataset^19^ presented here is the largest of its kind at this moment, assembled through the integration of domain-specific (Late Glacial archaeology and lithic technology) and computational expertise.

By linking original sources to coded data, we offer users the possibility to trace the material of interest back to source for validation. As our technical validation indicates, data availability and quality varies by region, creating an imbalance between Western Europe and the remainder of the study region. Especially in parts of Central and Eastern Europe, data availability is compromised by research historical factors, while taphonomic factors may have led to generally poorer preservation conditions in Northern Europe. In assembling the present dataset^19^, our aim has been to facilitate inter-regional analysis. Users seeking to ask questions at smaller geographic scales should be mindful of the regional integration of information within the toolkit and technology domains. This integration precludes comparisons of lithic technological variability between sites and at within-region scales. Furthermore, the 2D outline data derives from legacy sources and different production styles may impact accuracy^62^. Drawings and photos have, however, been shown to provide reliable information for geometric morphometric analysis^40,63^, lending confidence that these data can be used for comparative inter-regional analyses. Notably, the acquisition of additional outline data for lithic artefacts is straightforward, and we hope that users will complement the dataset^19^ at hand with further specimens. Our dataset^19^ provides the possibility of quantifying cultural similarity in an information-rich manner. In tandem with the increasing availability of palaeogenomic information, the dataset^19^ presented here thereby offers the opportunity to explicitly compare patterns of biological and cultural relatedness during this pivotal epoch in European prehistory, and therefore to contribute to general, discipline-transcending questions on early human biocultural evolution.

### Supplementary information

Supplementary Information

## Data Availability

The full dataset^19^ and the code used in this paper are freely available at Zenodo: 10.5281/zenodo.7940337. All data, analyses, and visualizations presented in this paper were prepared in R 4.2.2 under Ubuntu 18.04.5 LTS (64-bit) using the following R packages: *data.table* (≥1.14.8), *dplyr* (≥1.1.2), *forcats* (≥1.0.0), *ggforce* (≥0.4.1), *ggplot2* (≥3.4.2), *ggpointgrid* (≥1.2.0), *ggridges* (≥0.5.4), *magrittr* (≥2.0.3), *Momocs* (≥1.4.0), *outlineR* (≥0.1.0), *raster* (≥3.6–20), *readr* (≥2.1.4), *remotes* (≥2.4.2), *rgeos* (≥0.6–2), *rworldmap* (≥1.3–6), *sp* (≥1.6-0).
