# Supplementary material for: A pan-European dataset revealing variability in lithic technology, toolkits, and artefact shapes ~15-11 kya
Source: Sci Data. 2023 Sep 7;10:593. doi: 10.1038/s41597-023-02500-9 (PMC10484899; doi:10.1038/s41597-023-02500-9)
Supplement: Supplementary file 1 — Supplementary Information [file 41597_2023_2500_MOESM1_ESM.pdf]

Supplementary Information of Hussain *et al.* A pan-European dataset revealing variability in lithic technology, toolkits, and artefact shapes ~15-11 kya. *Sci. Data* (2023).

SI Part 1

Regional and site-level units of analysis

Supplementary Table 1. Overview of macro-regional units of analysis and their corresponding IDs and regional expert IDs (n=16).

Macroregional Unit	ID/Macro_region_code	Expert ID
Southern Scandinavia	SS	FR
Lithuania	LT	TR
Northern Germany	NG	DG
Britain	GBS	WM
Poland	PL	DS
Belgium and Southern Netherlands	BSN	HV, PC
Bohemia and Moravia	BOMO	MM
Southern Germany	SG	TH
Northern France	NF	LM, MB
Austria, Slovakia and Hungary	ASH	CP
Switzerland	CH (or SW)	TH
Northern/North-eastern Italy	NEI	FF
Western France	SWF	ML, NN
Cantabrian Spain	IBC	JFLdP
Mediterranean Iberia	IBM	JFLdP
Atlantic Iberia	IBA	JFLdP

Supplementary Table 2. Overview of total number of recorded key sites per macro-region (a full list of sites can be extracted from the original dataset published with this paper). Note that these numbers are not directly comparable since layer or concentration distinctions have sometimes been recorded as separate occurrence even when the larger site-context is identical, and sometimes not depending on the nature of the context: macro-regions marked with * involve site entries resolved on the level of excavation context or concentration; the actual number of archaeological sites is thus lower.

Macroregional Unit	Number of key sites
Southern Scandinavia	33
Lithuania	17
Northern Germany	19
Britain	40
Poland	83*

Belgium and Southern Netherlands	16
Bohemia and Moravia	12
Southern Germany	10
Northern France	9
Austria, Slovakia and Hungary	13
Switzerland	11
Northern/North-eastern Italy	13
(South)Western France	18
Cantabrian Spain	19
Mediterranean Iberia	30
Atlantic Iberia	7
total	**350**

Supplementary Table 3. Tabulation of recorded named archaeological cultures (NACs) ordered according to macro-region and arranged in equidistant time slices (TS I to IV).

Time-slice → <br>*Macro-region ↓*	***TS I***	***TS II***	***TS III***	***TS IV***
*Southern Scandinavia (**SS**)*	Havelte	Federmesser, Brommean	Brommean	Ahrensburgian, Maglemose
*Lithuania (**LT**)*		Hamburgian, Federmesser	Federmesser, Brommean, Ahrensburgian	Ahrensburgian
*Northern Germany (**NG**)*	Hamburgian, Federmesser	Havelte, Federmesser, Brommean	Federmesser, Ahrensburgian	Maglemose
*Britain (**GBS**)*	Creswellian/Late Magdalenian, Backed Blade or Hengistbury-type CBP, Havelte	Backed Blade or Hengistbury-type CBP, Havelte	Allerød CBP	Long Blade Industries/Epi-Ahrensburgian (FBBT), Early Mesolithic

*Poland (**PL**)*	Magdalenian, Hamburgian	Kamienna variant, Witowian	Witowian, Brommean, Perstunian, Wolkuszian	Wolkuszian, Epi-Ahrensburgian, Swiderian, Swiderian-Ahrensburgian, Pludian
*Belgium and Southern Netherlands (**BSN**)*	Magdalenian	Federmesser	(Epi-)Ahrensburgian	Epi-Ahrensburgian
*Bohemia and Moravia (**BOMO**)*	Magdalenian	Magdalenian, Epimagdalenian, ABP Federmesser, ABP Tishnovian	ABP Tishnovian	Mesolithic
*Southern Germany (**SG**)*	Late/Transitional Magdalenian	Late Palaeolithic	Late Palaeolithic, Atzenhofer group	Late Palaeolithic, Atzenhofer group, Beuronian A
*Northern France (**NF**)*	Upper Magdalenian, Early Azilian	Early Azilian, Late Azilian	Late Azilian	Belloisian
*Austria, Slovakia and Hungary (**ASH**)*	Late Magdalenian, Late Epigravettian	Aziloid Alps, Aziloid Tradition Eastern Central Europe	Aziloid Alps, Aziloid Tradition Eastern Central Europe, Late Palaeolithic	Aziloid Alps, Aziloid Tradition Eastern Central Europe, Late Palaeolithic, Recent Epigravettian
*Switzer-land (**CH**)*	Late Magdalenian/Technokomplex E, Older Azilian	Older Azilian, Fazies Fürsteiner	Older Azilian, Fazies Fürsteiner, Younger Azilian	Fazies Fürsteiner, Early Mesolithic I
*North-eastern Italy (**NEI**)*	Late Epigravettian (Phase 2)	Late Epigravettian (Phase 2), Late Epigravettian (Phase 3)	Late Epigravettian (Phase 3)	Late Epigravettian-Early Sauveterrian, Early Sauveterrian
*Western France (**SWF**)*	Upper Magdalenian, Early Azilian	Early Azilian, Late Azilian	Late Azilian, Early Laborian	Early Laborian, Late Laborian
*Cantabrian Spain (**IBC**)*	Final Magdalenian	Azilian	Azilian	Azilian

Mediter-ranean Iberia ***(IBM)***	Upper Magdalenian, Final Magdalenian, Azilian	Azilian, Epipalaeolithic/Epimagdalenian	Azilian, Epipalaeolithic/Epimagdalenian, Sauveterrian/Sauveterroid Epipalaeolithic	Sauveterrian/Sauveterroid Epipalaeolithic
Atlantic Iberia ***(IBA)***	Upper Magdalenian	Final Magdalenian	Azilian	Azilian

SI Part 2

Site quality

Data quality is assessed on the level of individual archaeological sites and presented as a 'Quality_Score' (see main text and **Supplementary Table 6** below).

Supplementary Table 4. Quality score per region per time-slice.

Macroregion-code and number of observations	**Discrete time-slice**	**Quality score (median)**
ASH (n=5)	I	5
BSN (n=4)	I	4
BOMO (n=3)	I	4
NEI (n=3)	I	4
NG (n=6)	I	4
SG (n=3)	I	4
CH (n=5)	I	4
SWF (n=7)	I	4
PL (n=8)	I	4
IBC (n=7)	I	6
IBM (n=10)	I	5
IBA (n=2)	I	6
SS (n=6)	I	3.5
NF (n=5)	I	5
GBS (n=18)	I	3.5
ASH (n=4)	II	2.5
BSN (n=5)	II	4

BOMO (n=8)	II	3
LT (n=4)	II	0
NEI (n=5)	II	4
NG (n=9)	II	3
CH (n=4)	II	4
SG (n=1)	II	4
SWF (n=6)	II	3
PL (n=17)	II	4
IBC (n=6)	II	5
IBM (n=4)	II	6
IBA (n=1)	II	6
SS (n=8)	II	3
NF (n=4)	II	5
GBS (n=4)	II	3.5
SS (n=9)	III	3
ASH (n=4)	III	2
BSN (n=4)	III	4
BOMO (n=3)	III	3
LT (n=8)	III	0
NEI (n=4)	III	5.5
NG (n=7)	III	4
CH (n=4)	III	3.5
SG (n=5)	III	4

SWF (n=6)	III	3
PL (n=29)	III	4
IBC (n=6)	III	5
IBM (n=6)	III	5
IBA (n=2)	III	6
NF (n=2)	III	5
GBS (n=11)	III	4
ASH (n=6)	IV	3.5
BSN (n=3)	IV	3
BOMO (n=1)	IV	1
LT (n=6)	IV	3.5
NEI (n=5)	IV	5
NG (n=4)	IV	5
CH (n=3)	IV	4
SG (n=5)	IV	3
SWF (n=7)	IV	5
PL (n=29)	IV	3
IBC (n=4)	IV	4.5
IBM (n=5)	IV	6
IBA (n=2)	IV	5
SS (n=10)	IV	4
NF (n=2)	IV	5.5
GBS (n=11)	IV	5

<u>SI Part 3</u>

The 1511NAC Dataset

Metadata

Supplementary Table 5. General abbreviations (provided as numbers and as text entries).

Code	Description
KeySite	Name of selected key site
Level/layer/ concentration	Context of selected lithic assemblage from key site
Site_ID	Unique site identifier
TaxUnit	Name of associated archaeological taxonomic unit
TaxUnit_unique	Unique TaxUnit identifier
Timeslice	Time-slice attribution (“1”, “2”, “3” or “4”); if a given TaxUnit spans multiple Timeslices, these a listed as comma separated numbers (e.g. “1,2”)
Expert_editor	Code for data provider
Macro_region	Macro-region for which lithic data was collected (frame of reference for TaxUnit determination)
Macro_region_code	Unique identifier of macro-regions

Site/context data module

In contrast to the previous two data modules, the site/context module compiles categorical and non-categorical information on the level of individual key sites chosen to represent larger taxonomic archaeological units. Contextual information is either given as numbers, text, a combination of the two, or as predefined categorical selections (drop-down).

Supplementary Table 6. Calculated site-quality scores

Code	Description
Quality_Score	Total sum of following scores: ‘1’ for Dating_qual = ‘Reliable’, ‘1’ for Site_strat = ‘Stratified’, ‘1’ for Ass_pos = ‘Primary/insitu’, ‘1’ for Ass_coh = ‘Homogeneous’ + ‘1’ for Site_excav = ‘1980-2000’ or ‘2’ for Site_excav = ‘After 2000’ (total possible score = ‘6’)
Quality_Rank	‘1’ if Quality_Score = 5-6, ‘2’ if Quality_Score = 3-4, ‘3’ if Quality_Score = 1-2 or ‘4’ if Quality_Score = 0

Supplementary Table 7. Register of processed and recorded tool outlines (provided as ‘1’ for available and ‘0’ for not available).

Code	Description
Outline_AR_TS1	Armature outlines for Timeslice 1
Outline_AR_TS2	Armature outlines for Timeslice 2
Outline_AR_TS3	Armature outlines for Timeslice 3
Outline_AR_TS4	Armature outlines for Timeslice 4
Outline_ES_TS1	Endscraper outlines for Timeslice 1
Outline_ES_TS2	Endscraper outlines for Timeslice 2
Outline_ES_TS3	Endscraper outlines for Timeslice 3
Outline_ES_TS4	Endscraper outlines for Timeslice 4
Outline_BR_TS1	Borer/perforator outlines for Timeslice 1
Outline_BR_TS2	Borer/perforator outlines for Timeslice 2
Outline_BR_TS3	Borer/perforator outlines for Timeslice 3
Outline_BR_TS4	Borer/perforator outlines for Timeslice 4

SI Part 4

Outline library

Data structure and recording scheme

The outline dataset consists of individual image files, which have been pre-processed into binary artefact images, and which can directly be fed into an outline extraction and shape-description workflow (see detailed description of extraction and data preparation methods in the main paper). Original artefact imagery does not necessarily contain only a single lithic artefact but sometimes gather multiple objects. This being said, the automated extraction protocol used generates individual files for each extracted object-outline (Matzig 2021a, b). It is these individual object-outline image files (images as data), which are stored as JPEG files in the dataset. Fully pre-processed artefact outlines are also made available and are stored separately in the dataset.

Individual outlines are grouped and coded according the three main tool categories structuring the outline sample (Armature = 'AR'; Endscraper = 'ES'; Borer/perforator = 'BR'). Individual files/images are linked by naming conventions (see below) reflecting their associated time slice, taxonomic unit, site, literature source, etc.

Note that for the purpose of our research goals/interests, these broader categories (AR, ES, BR) were not further disintegrated into individual lithic types, morphotypes or techno-types that are commonly distinguished in the literature.

Furthermore, only complete or reconstructed pieces have been included in the outline dataset for lithic armatures (AR) and endscrapers (ES), but not for borers/perforators (BR) because in the latter case the analytical aim of the data collection was not to analyse whole-outline variability but the shape configuration of borer working-ends (open outlines).

This is the general structure of the employed naming convention:

- **Timeslice_TaxonomicUnit_Macroregion_KeySite_LiteratureSource_ToolCat_Axiality_SubID1(_SubID2)**

Supplementary Table 8. Break-down of the different parts of this label structure and how they are expressed in the naming of individual image files.

Timeslice	'TS1', 'TS2', 'TS3' or 'TS4' for Timeslice 1, 2, 3 or 4; image data that relates to multiple Timeslices is coded in the following way: Timeslices 1-2 = 'TS12', Timeslices 3-4 = 'TS34' or Timeslices 2-4 = 'TS234'
TaxonomicUnit	Identifier taken from 'TaxUnit_unique' (see Metadata)
Macroregion	Identifier taken from 'Macro_region_code' (see Metadata)
KeySite	Identifier taken from 'Site_ID' (see Metadata)
LiteratureSource	Short literature reference without spaces, e.g., 'Mayer2000'; in case of multiple authors, e.g., 'WebsterHut1998' or 'Greenwoodetal2003'
ToolCat	Code for overarching/main tool categories mentioned above: 'AR' for Armature, 'ES' for Endscrapers, and 'BR' for Borers/perforators (including *Zinken*)

Axiality	Axiality of points/orientation of tool axis in relation to blank axis: ‘d’ = distal point (point axis aligns with blank axis), ‘p’ = proximal point (point axis inversed in relation to blank axis), or ‘na’ = not applicable/not determinable
SubID1	Sub-id assigned to image files in numerical order that share all previous information
SubID2	Sub-id assigned to individualized object-outlines in numerical after automated outline extraction

References

Matzig, D.N. 2021a. outlineR: An R package to derive outline shapes from (multiple) artefacts on JPEG images. *Zenodo*. https://doi.org/10.5281/ZENODO.4527469.

Matzig, D.N. 2021b. *outlineR: Artefact Processing and Extraction Protocol v1*. DOI:dx.doi.org/10.17504/protocols.io.bygaptse (external link: https://www.protocols.io/view/outliner-artefact-processing-and-extraction-protoc-rm7vz3pp8gx1/v1 09/22/2021).
